# Direct DNA and RNA detection from large volumes of whole human blood

**DOI:** 10.1038/s41598-018-21224-0

**Published:** 2018-02-21

**Authors:** Dongyang Cai, Ole Behrmann, Frank Hufert, Gregory Dame, Gerald Urban

**Affiliations:** 1 Department of Microsystems Engineering, University of Freiburg, Freiburg, DE-79110 Germany; 2 Department of Microbiology and Virology, Brandenburg Medical School Fontane, Senftenberg, DE-01968 Germany

## Abstract

PCR inhibitors in clinical specimens negatively affect the sensitivity of diagnostic PCR and RT-PCR or may even cause false-negative results. To overcome PCR inhibition, increase the sensitivity of the assays and simplify the detection protocols, simple methods based on quantitative nested real-time PCR and RT-PCR were developed to detect exogenous DNA and RNA directly from large volumes of whole human blood (WHB). *Thermus thermophilus (Tth)* polymerase is resistant to several common PCR inhibitors and exhibits reverse transcriptase activity in the presence of manganese ions. In combination with optimized concentrations of magnesium ions and manganese ions, *Tth* polymerase enabled efficient detection of DNA and RNA from large volumes of WHB treated with various anticoagulants. The applicability of these methods was further demonstrated by examining WHB specimens collected from different healthy individuals and those stored under a variety of conditions. The detection limit of these methods was determined by detecting exogenous DNA, RNA, and bacteria spiked in WHB. To the best of our knowledge, direct RNA detection from large volumes of WHB has not been reported. The results of the developed methods can be obtained within 4 hours, making them possible for rapid and accurate detection of disease-causing agents from WHB.

## Introduction

PCR- and RT-PCR-based diagnostic assays may have low sensitivity or even false-negative results when PCR inhibitors are present in clinical specimens^1^. Whole human blood (WHB) is of particular interest as a clinical specimen because of its ease of collection and availability of large volumes. It has been comprehensively used for PCR- and RT-PCR-based diagnosis of bacterial and viral infections, hereditary disease and forensic analysis^2–5^. More recently, several studies have shown that circulating nucleic acids in plasma and serum allow the prognosis, diagnosis, and monitoring of various diseases such as cancer, trauma and prenatal disease^6–8^. However, either innate components of WHB including immunoglobulin G, hemoglobin, lactoferrin, leukocyte DNA or added anticoagulants such as EDTA, citrate, and heparin have been identified as PCR inhibitors^9–14^. *Taq* DNA polymerase and Ampli*Taq* Gold, the most commonly used polymerases for PCR, can be completely inhibited in the presence of less than 0.2% WHB^15,16^. PCR inhibitors generally exert their effects through inactivation of DNA polymerases, binding of DNA polymerase co-factors or degradation of target nucleic acids and/or primers^1^. Fewer PCR inhibitors exist in plasma and serum than WHB, but the detection rate of some causative pathogens in such specimens can be lower than that from WHB due to the loss of target^3,17^.

Various methods for DNA and RNA extraction from WHB have been developed^18–20^. However, these methods are generally time-consuming and labor-intensive and increase cost. Furthermore, the multiple sample processing steps involved in these methods increase the risk of cross-contamination and lead to the loss of target^10^. In addition, some PCR inhibitors still exist even after DNA and RNA extraction^1^.

An alternative to DNA and RNA extraction is direct nucleic acid detection from WHB. Previous investigators have reported several methods for WHB DNA detection. However, sample pretreatment involving preheating^21^, alternating heating-cooling^22^, and freezing-thawing^23^ of WHB were needed, which were still time-consuming and cumbersome. Furthermore, various additives such as bovine serum albumin (BSA), dimethyl sulphoxide, non-ionic detergents, bacteriophage T4 gene 32 protein, proteinase inhibitors^15^ and PCR enhancer cocktail^24^, as well as specific PCR buffers such as Anydirect PCR buffer^25^, Ampdirect PCR buffer^26^, and high pH PCR buffer^27^ were used in WHB DNA detection. In addition, mutant forms of *Taq* DNA polymerase have been developed for direct DNA detection from large volumes of WHB^28^.

Fewer studies have been reported concerning direct RNA detection from WHB. This is probably because the fragility of RNA and the existence of high levels of RNases which can cause RNA degradation and compromise RNA integrity. One method involving a sample pretreatment step to remove erythrocytes and lyse leukocytes has been reported^29^. However, the blood specimen used in that work after sample pretreatment was no longer WHB. In other studies, direct nested RT-PCR has been reported for the detection of bovine viral diarrhoea virus from whole bovine blood^30,31^. However, the maximum volume of whole bovine blood that could be detected directly was still limited (2% of total RT-PCR volume). To the best of our knowledge, direct RNA detection from large volumes of WHB has not been reported.

The aim of this study is to develop methods to detect exogenous DNA and RNA directly from large volumes of WHB without the need for sample pretreatment, additives or specific PCR buffers. *Tth* polymerase, a thermostable enzyme isolated from eubacterium *Thermus thermophilus* strain HB8 and expressed in *Escherichia coli* (*E*. *coli*), can tolerate high concentrations of inhibitory substances present in clinical specimens^32–34^. This enzyme also exhibits reverse transcriptase activity in the presence of manganese ions^35^. The effects of magnesium ions and manganese ions on the ability of *Tth* polymerase to detect DNA and RNA from WHB were investigated. Additionally, the effects of four preanalytical factors: (a) WHB treated with different anticoagulants, (b) WHB collected from different individuals, (c) storage of WHB at different temperatures and (d) time delay in blood processing after collection on the ability of *Tth* polymerase to detect DNA and RNA from WHB were also investigated. Finally, the ability of *Tth* polymerase to detect low amounts of exogenous DNA, RNA, and bacteria spiked in WHB were also studied. The developed methods were evaluated using quantitative nested real-time PCR and RT-PCR because of the following reasons: a) since real-time PCR and RT-PCR were used, inhibitory effects could be measured by the increase in quantification cycle (Cq) value; b) the sensitivity of *Tth* polymerase has been reported to be two orders of magnitude lower than that of *Taq* polymerase for RNA detection^36^, nested RT-PCR provided higher analytical sensitivity; c) the formation of an opaque precipitate after WHB PCR and RT-PCR blocked the fluorescence detection and the mixtures became increasingly turbid as the blood concentration increased. Nested real-time PCR using the first round PCR and RT-PCR amplicons as template circumvented this issue.

## Results

### Effects of magnesium ions and manganese ions concentrations on DNA and RNA detection from EDTA treated WHB

**Figure 1:**
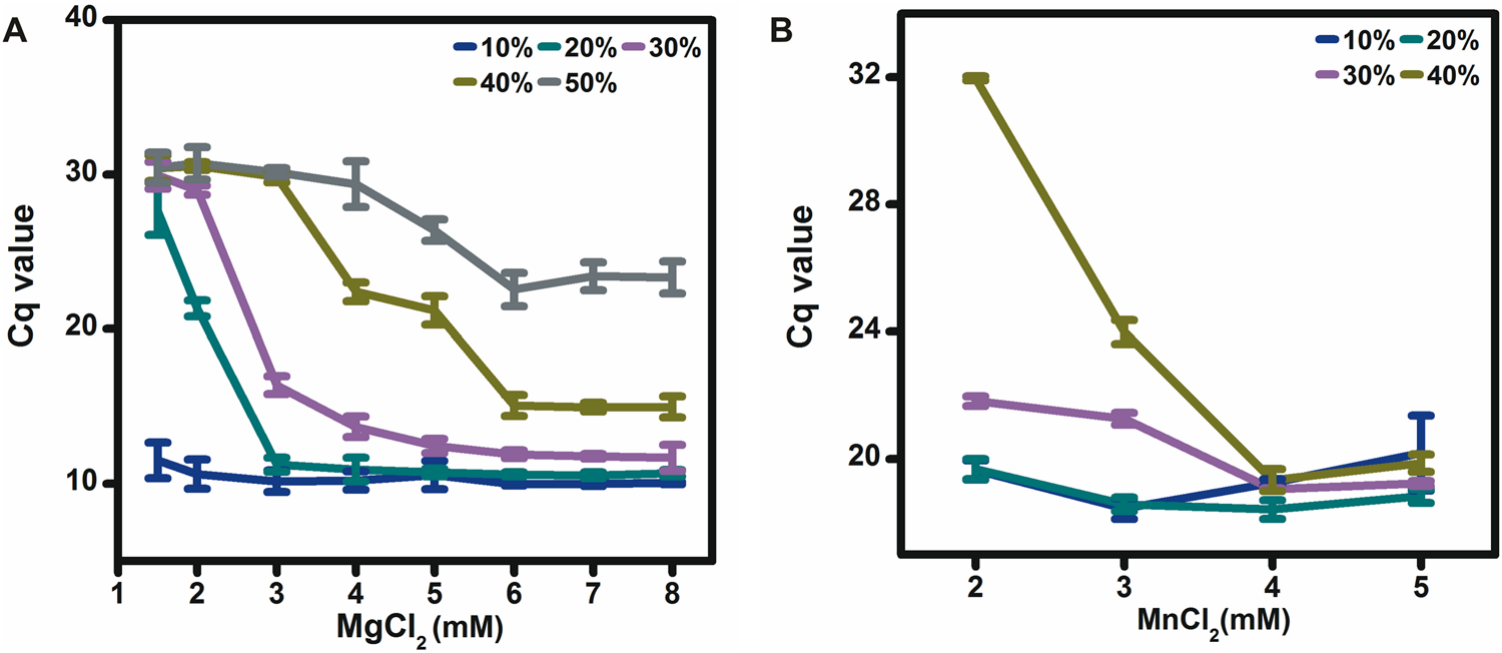
The Cq values for DNA (**A**) and RNA (**B**) detection from varying amounts of EDTA treated WHB in the presence of different concentrations of magnesium ions and manganese ions.

Magnesium ions and manganese ions are required co-factors for *Tth* polymerase and adequate concentrations of these ions are crucial for successful DNA and RNA detection. Different concentrations of magnesium ions (from 1.5 mM to 8 mM) and manganese ions (from 2 mM to 5 mM) were used in the first round PCR and RT-PCR reactions to detect the *CNRZ16* gene and tmRNA from varying amounts of EDTA treated WHB. The amplification curves of these experiments are shown in Figure S1 and the Cq values for these experiments are summarized in line graphs as shown in Fig. 1. We defined that when the Cq no longer shifted to lower values (<1 Cq value) with increased concentrations of magnesium ions or manganese ions, these concentrations of ions were adequate for the corresponding concentrations of WHB. In this situation, the adequate concentrations of magnesium ions for DNA detection from 10%, 20%, 30%, 40% and 50% WHB were 1.5, 3, 5, 6, and 6 mM, respectively (the mean Cq value for NCC minus 3 Cq values was 28.68). The adequate concentrations of manganese ions for RNA detection from 10%, 20%, 30%, and 40% WHB were 3, 3, 4, and 4 mM, respectively. 6 mM magnesium ions and 4 mM manganese ions were adequate for all concentrations of WHB tested and were chosen for the following DNA and RNA detection experiments. The specificity of *Tth* polymerase for DNA detection in the presence of 6 mM magnesium ions and RNA detection in the presence of 4 mM manganese ions was demonstrated by gel electrophoresis of the first round PCR and RT-PCR products as shown in Figure S2. No unspecific PCR or RT-PCR products were observed from the gels.

### Effects of citrate and heparin on DNA and RNA detection from WHB

**Figure 2:**
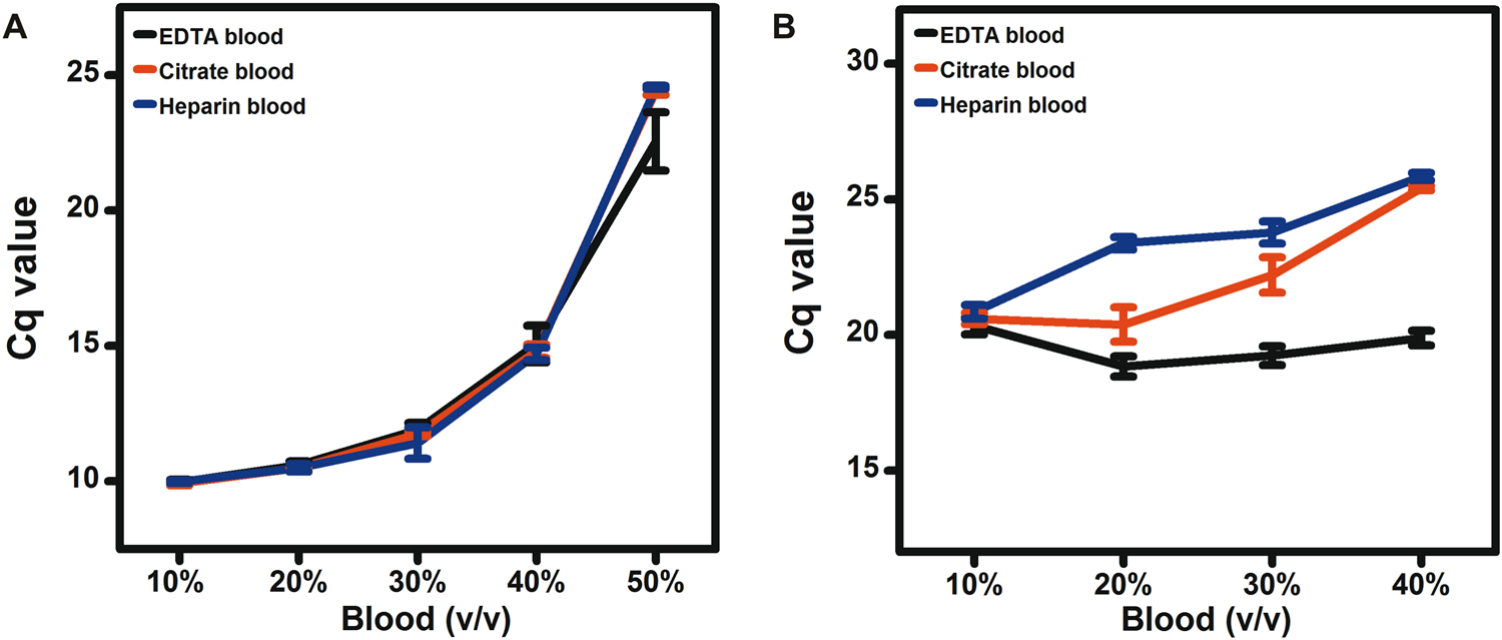
The Cq values for DNA (**A**) and RNA (**B**) detection from varying amounts of WHB treated with EDTA, citrate and heparin in the presence of 6 mM magnesium ions and 4 mM manganese ions.

EDTA, citrate, and heparin are the most commonly used anticoagulants in clinical hematology. The effects of citrate and heparin on the ability of *Tth* polymerase to detect DNA and RNA from varying amounts of WHB in the presence of adequate concentrations of magnesium ions and manganese ions were evaluated. The Cq values for these experiments are summarized in line graphs as shown in Fig. 2. These results indicated that *Tth* polymerase enabled DNA and RNA detection from various anticoagulants treated WHB in the presence of adequate concentrations of magnesium ions and manganese ions. We also found that the Cq values for DNA detection from 50% EDTA treated WHB were significantly smaller than those from 50% citrate and heparin treated WHB (p < 0.01) as shown in Fig. 2A, meanwhile the Cq values for RNA detection from 20–40% EDTA treated WHB were significantly smaller than those from 20–40% citrate and heparin treated WHB (p < 0.01) as shown in Fig. 2B. Therefore, EDTA would be the preferred anticoagulant for DNA and RNA detection from large volumes of WHB. EDTA treated WHB was chosen for the following DNA and RNA detection experiments.

### Effects of WHB collected from different individuals on DNA and RNA detection

**Figure 3:**
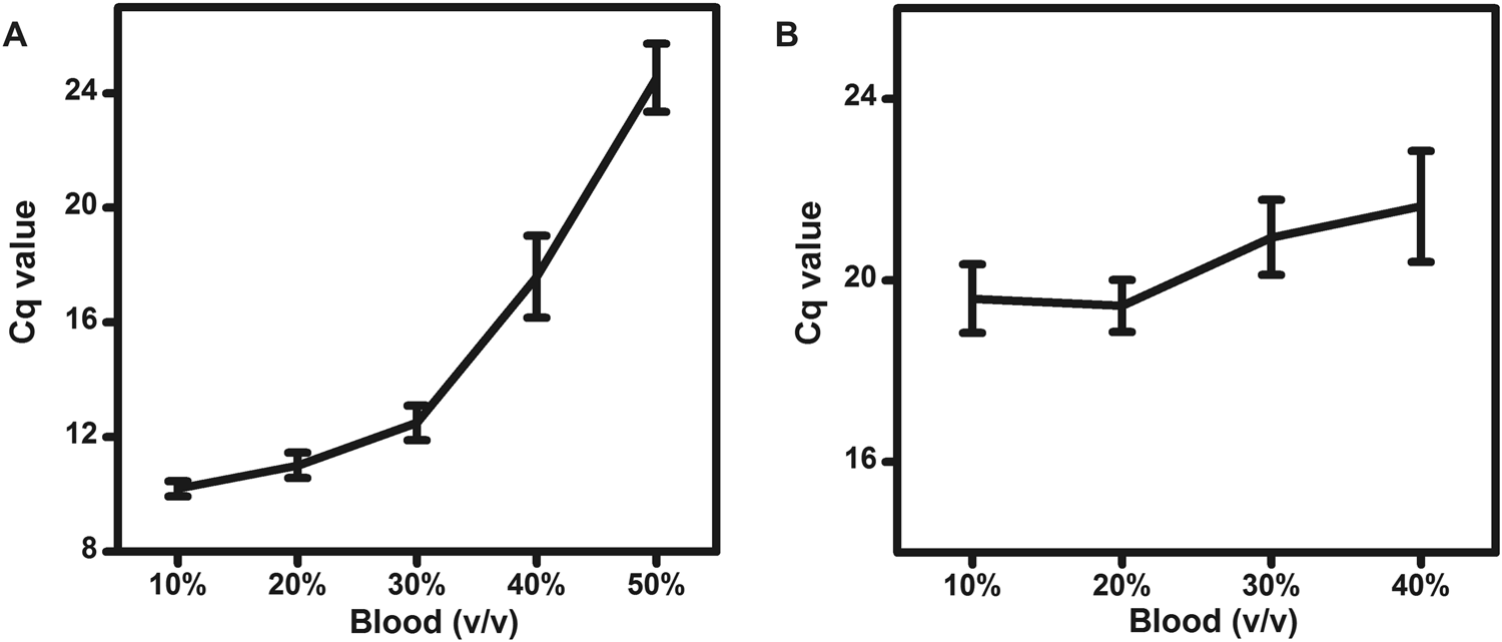
The Cq values for DNA (**A**) and RNA (**B**) detection from varying amounts of WHB collected from 10 healthy individuals in the presence of 6 mM magnesium ions and 4 mM manganese ions.

Clinical WHB specimens collected from different individuals contain a variety of substances at different concentrations. The effects of WHB collected from 10 individuals on the ability of *Tth* polymerase to detect DNA and RNA in the presence of adequate concentrations of magnesium ions or manganese ions were evaluated. The Cq values for these experiments are summarized in line graphs as shown in Fig. 3. From these results, we tentatively concluded that the combination of *Tth* polymerase with adequate concentrations of magnesium ions and manganese ions was capable of reproducibly detecting DNA and RNA from WHB specimens collected from different individuals. We also found that the Cq values for RNA detection from 10% to 20% EDTA treated WHB remained nearly unchanged as shown in Fig. 3B. These results indicated that the combination of *Tth* polymerase with adequate concentrations of manganese ions enabled RNA detection from 10–20% WHB without reduced detection capacity.

### Effects of WHB stored under a variety of conditions on DNA and RNA detection

**Figure 4:**
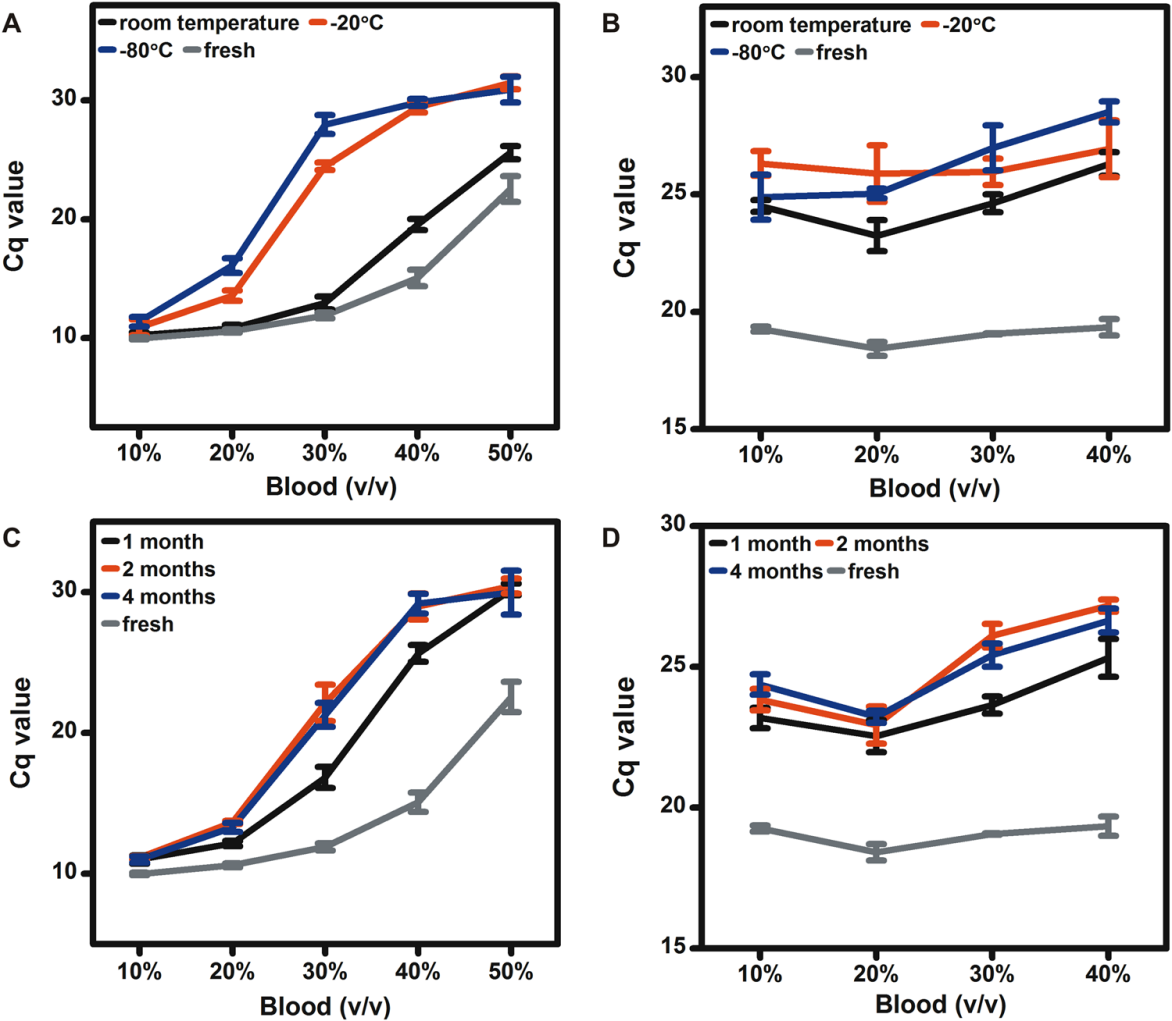
The Cq values for DNA (**A**) and RNA (**B**) detection from varying amounts of WHB stored at room temperature, −20 °C and −80 °C for ten days and those from fresh WHB in the presence of 6 mM magnesium ions and 4 mM manganese ions; the Cq values for DNA (**C**) and RNA (**D**) detection from varying amounts of WHB stored at 4 °C for 1, 2 and 4 months and those from fresh WHB in the presence of 6 mM magnesium ions and 4 mM manganese ions.

Depending on the aim of laboratory analysis, WHB and blood fractions may be stored under a variety of conditions. The effects of WHB stored at room temperature, −20 °C and −80 °C for ten days on the ability of *Tth* polymerase to detect DNA and RNA in the presence of adequate concentrations of magnesium ions or manganese ions were evaluated. The Cq values for these experiments as well as those from fresh WHB are shown in Fig. 4A and B. These results indicated that not only fresh blood, but also WHB specimens that have been stored at room temperature or in frozen states for a minimum of 10 days could be analyzed by *Tth* polymerase in combination with adequate magnesium ions and manganese ions. We also found that the Cq values for DNA and RNA detection from WHB stored at room temperature, −20 °C, and −80 °C for ten days shifted to higher values compared to those from fresh WHB (p < 0.01) as shown in Fig. 4A and B.

The effects of WHB stored at + 4 °C for 1, 2 and 4 months on the ability of *Tth* polymerase to detect DNA and RNA in the presence of adequate concentrations of magnesium ions or manganese ions were also evaluated. The Cq values for these experiments as well as those from fresh WHB are shown in Fig. 4C and D. These results indicated that WHB specimens that have been stored in cold condition for different storage durations as long as 4 months could be analyzed by *Tth* polymerase in combination with adequate magnesium ions and manganese ions. We also found that the Cq values for DNA and RNA detection from varying amounts of WHB stored at + 4 °C for different storage durations shifted to higher values compared to those from fresh WHB (p < 0.01). Among these three storage durations, the Cq values for DNA detection from 20–50% WHB stored at 4 °C for 2 and 4 months were much higher than those from WHB stored at 4 °C for 1 month (p < 0.01) as shown in Fig. 4C, meanwhile the Cq values for RNA detection from 10%, 30%, and 40% WHB stored at 4 °C for 2 and 4 months were much higher than those from WHB stored at 4 °C for 1 month (p < 0.01) as shown in Fig. 4D.

### Detection limit of DNA, RNA, and bacteria from varying amounts of WHB

**Figure 5:**
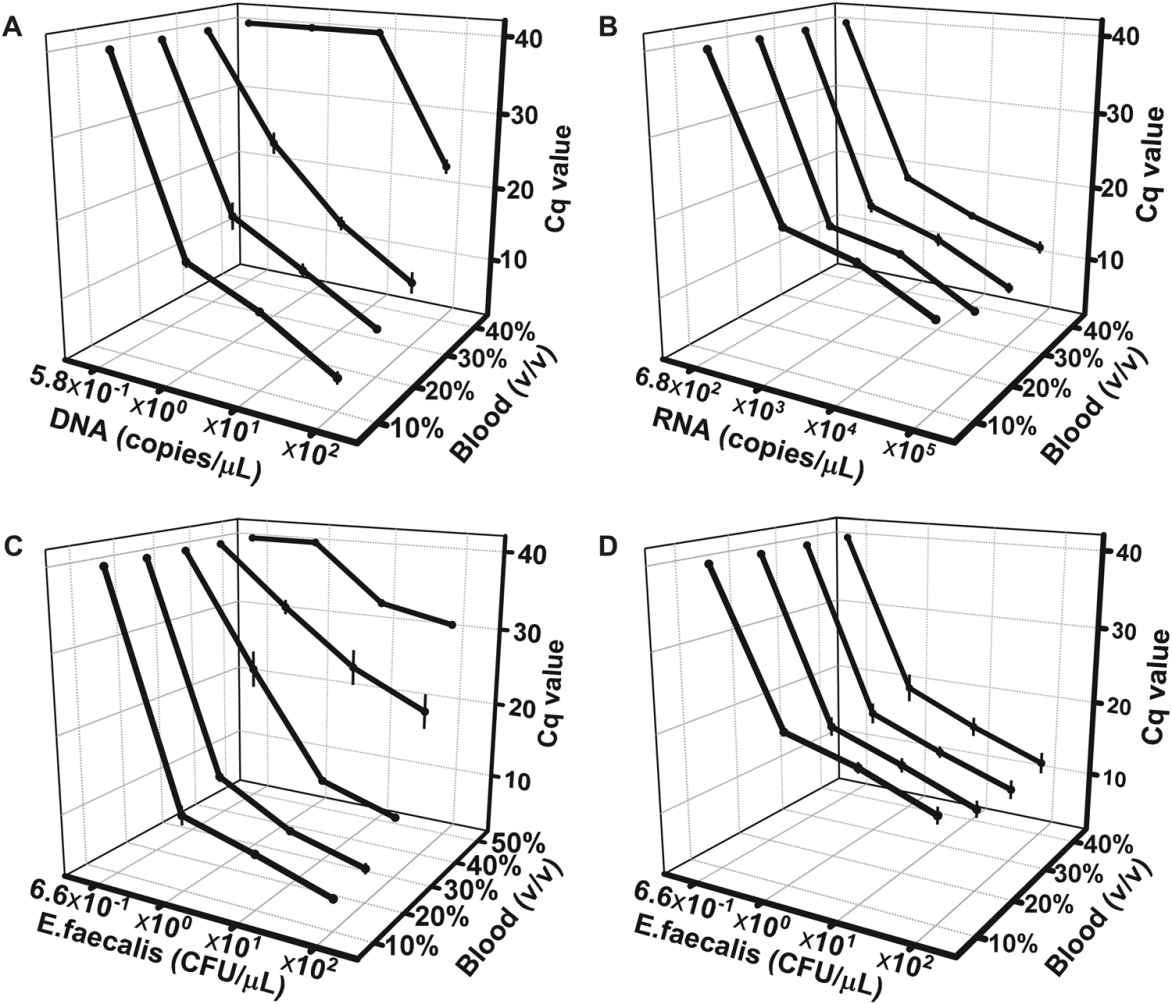
The Cq values for low amounts of DNA (**A**) and RNA (**B**) detection from varying amounts of WHB in the presence of 6 mM magnesium ions and 4 mM manganese ions; the Cq values for the detection of low amounts of bacteria targeting the *CNRZ16* gene (**C**) and tmRNA (**D**) from varying amounts of WHB in the presence of 6 mM magnesium ions and 4 mM manganese ions.

The ability of *Tth* polymerase to detect low concentrations of DNA and RNA from varying amounts of WHB in the presence of adequate concentrations of corresponding ions was evaluated. A 10-fold dilution series of *E*. *faecalis* Symbioflor 1 genomic DNA using EDTA treated WHB was prepared to mimic a clinical specimen. Failure of the reaction to detect DNA is represented in the graph by a Cq value of 40. The detection limit of *E*. *faecalis* Symbioflor 1 genomic DNA from 10%, 20%, and 30% WHB was 5.8 copies/μL as shown in Fig. 5A. Whereas the detection limits from 40% and 50% WHB were reduced by 2 log units (5.8 × 10^2^ copies/μL) and at least 3 log units (no detection was observed with the given DNA dilutions, data not shown), respectively. As the exogenous DNA was suspended in WHB, more templates were available to *Tth* polymerase in the presence of higher concentrations of WHB. However, more DNA templates did not relieve the inhibitory effects caused by higher concentrations of WHB which led to significantly increased Cq values from 40% WHB (p < 0.01) as shown in Fig. 5A. Due to the existence of high levels of RNases in WHB which can cause RNA degradation and compromise RNA integrity, tmRNA can not be suspended or diluted with WHB. To ensure that more tmRNA was introduced to RT-PCR mixtures with higher concentrations of WHB used, four 10-fold dilution series of tmRNA corresponding to 10%, 20%, 30%, and 40% WHB by DEPC-treated water were prepared. The equivalent concentrations of tmRNA in each concentration of WHB were from 6.8 × 10^5^ to 6.8 × 10^2^ copies/μL. The detection limit of *E*. *faecalis* Symbioflor 1 tmRNA from 10%, 20%, 30%, and 40% WHB was 6.8 × 10^3^ copies/μL as shown in Fig. 5B. The Cq values for tmRNA detection from different concentrations of WHB tested were similar.

Since the developed methods should also serve to the diagnosis of bacterial infections, the detection limit of bacteria was also evaluated. Three times washing using sterile saline was used to remove the nucleic acids attached to the bacteria. A dilution series of *E*. *faecalis* Symbioflor 1 using EDTA treated WHB was prepared to mimic a clinical specimen. The detection limit of *E*. *faecalis* Symbioflor 1 targeting the *CNRZ16* gene from 10%, 20%, 30%, and 40% WHB was 6.6 CFU/μL as shown in Fig. 5C. Whereas the detection limit from 50% WHB was reduced by 1 log units (66 CFU/μL). More targets introduced into the PCR mixtures did not relieve the inhibitory effects caused by higher concentrations of WHB which led to significantly increased Cq values from 40% and 50% WHB (p < 0.01). The detection limit of *E*. *faecalis* Symbioflor 1 targeting tmRNA from 10%, 20%, 30%, and 40% WHB was also 6.6 CFU/μL as shown in Fig. 5D. The Cq values for bacterial detection targeting tmRNA from different concentrations of WHB tested were similar.

## Discussion

PCR- and RT-PCR-based diagnostic assays of WHB may have low sensitivity or even false-negative results caused by PCR inhibitors. *Tth* polymerase has proven to be resistant to several common PCR inhibitors in clinical specimens^32–34^, and exhibits reverse transcriptase activity in the presence of manganese ions^35^. There is also evidence that RNA detection by *Tth* polymerase is more resistant to inhibitors present in nasopharyngeal swab compared to *Taq* polymerase^36^. Therefore, we attempted to use *Tth* polymerase to directly detect DNA and RNA from large volumes of WHB without the need for sample pretreatment, additives or specific PCR buffers.

We found that when supplied PCR buffer containing 1.5 mM magnesium ions was used in WHB PCR reactions, *Tth* polymerase enabled DNA detection only from 10% WHB (Fig. 1A). Moreover, when 2 mM manganese ions were used in WHB RT-PCR reactions, *Tth* polymerase enabled RNA detection from 40% WHB with increased Cq values (Fig. 1B). EDTA is commonly used in WHB as anticoagulant to prevent clotting and maintain specimen quality. It becomes operative through binding of calcium ions in WHB which is necessary for a variety of enzyme reactions of the coagulation cascade^37^. However, excessive EDTA can become a PCR inhibitor through binding of DNA polymerase co-factors (magnesium ions and manganese ions), especially in the reactions with high concentrations of WHB. Therefore, the concentrations of magnesium ions and manganese ions in WHB PCR and RT-PCR reactions should be increased to compensate for the inhibitory effect of EDTA. After adding adequate concentrations of magnesium ions and manganese ions, *Tth* polymerase enabled DNA detection from 50% WHB, and RNA detection from 40% WHB with lower Cq values. The Cq values for DNA detection from varying amounts of WHB in the presence of 6, 7 and 8 mM magnesium ions were similar and those for RNA detection from varying amount of WHB in the presence of 4 and 5 mM manganese ions were similar (Fig. 1). Excessive magnesium ions and manganese ions may cause the fidelity of *Tth* DNA polymerase to be reduced and may cause the generation of unwanted products. Therefore, 6 mM magnesium ions and 4 mM manganese ions were chosen for the DNA and RNA detection experiments.

Laboratory tests are also performed on WHB treated with citrate and heparin anticoagulants. Citrate and heparin become operative through binding of calcium ions or by inhibiting thrombin activity, respectively. Excessive citrate can also become a PCR inhibitor in the same way as EDTA. The inhibitory effect of heparin has been reported on the basis of an interaction with DNA and DNA polymerase. Although both DNA and heparin are highly negatively charged, and would not be expected to interact, binding between these two molecules could be mediated by divalent cations such as magnesium ions^38^. Several studies have been reported on the inhibitory effects of various anticoagulants in different PCR reactions. In the case of detecting *Streptococcus pneumoniae* from blood, Friedland *et al*. observed PCR inhibitory effects in the presence of EDTA and citrate rather than heparin^13^. In the case of detecting *Aspergillus fumigatus* from plasma, Garcia *et al*. observed PCR inhibitory effects in the presence of citrate and heparin rather than EDTA^39^. In our study, the combination of *Tth* polymerase with adequate concentrations of magnesium ions and manganese ions can overcome the inhibitory effects of various anticoagulants in WHB. EDTA was likely to be the preferred anticoagulant for WHB DNA and RNA detection compared to citrate and heparin (Fig. 2). The higher concentration of citrate in WHB (9.6 mM) than that of EDTA (3.9 mM), and the multiplex inhibitory effects of heparin on DNA, DNA polymerase and DNA polymerase co-factors may explain the higher resistance of *Tth* polymerase to EDTA treated WHB for DNA and RNA detection.

We observed variations in the Cq values for DNA and RNA detection from fresh WHB, frozen WHB and WHB stored in cold condition for different durations. It is believed that a freezing-thawing cycle ruptures erythrocytes and leukocytes very efficiently due to the intracellular ice formation and probably to some extent due to hypertonicity^40^. The rupture of erythrocytes and leukocytes also happens to WHB specimens stored in cold condition which increases with the duration of storage. Meanwhile, the cells in WHB will eventually completely lyse anyway during denaturation step in PCR or RT-PCR^24^. We speculate that with prelysed blood, hemin formed by heme oxidation *in vitro* after erythrocyte rupture and exposure to the air, is mainly responsible for the observed variations in the Cq values for DNA and RNA detection from fresh WHB, frozen WHB and WHB stored in cold condition for different durations. Hemin has been reported to be inhibitory to DNA polymerases from human neuroblasoma cells and erythroid hyperplastic bone marrow cells and also reverse transcriptase from Rauscher murine leukemia virus^41–43^.

TmRNA was discovered in *E*. *coli* and described as small stable RNA, present at ~1000 copies per cell^44^. It was found that the detection limit of bacteria targeting genomic DNA (1 copy per cell, detection limit: 5.8 copies/μL) or tmRNA (~1000 copies per cell, detection limit: 6.8 × 10^3^ copies/μL) from 10% to 30% WHB was the same as shown in Fig. 5A and B. The detection limit of bacteria targeting tmRNA was 2 log units higher than that targeting genomic DNA from 40% WHB. The binding or degradation of low amounts of genomic DNA by inhibitory substances in high concentrations of WHB may explain this phenomenon. This was further proven by detecting intact bacteria targeting genomic DNA from 40% and 50% WHB as shown in Fig. 5C.

In summary, we developed simple methods to directly detect DNA and RNA from large volumes of WHB without the need for sample pretreament, additives or specific PCR buffers. The results of these methods can be obtained within 4 hours, making them possible for rapid and accurate detection of disease-causing agents from WHB.

## Materials and Methods

### Blood specimens

WHB specimens treated with tripotassium EDTA, trisodium citrate and sodium heparin were obtained from healthy individuals in the University Medical Center Freiburg, Germany. The WHB specimens were approved by the Research Ethics Committee of the University of Freiburg and the study was performed in accordance with the relevant guidelines. Informed consent was obtained from all the individuals. The citrate and heparin treated WHB specimens were stored at + 4 °C until use. The EDTA treated WHB specimens were split in half. One half was stored at + 4 °C, the other half was divided into three fractions and stored at room temperature (25 °C), −20 °C and −80 °C for ten days, respectively.

### Bacterial culture

*Enterococcus faecalis* Symbioflor 1 (*E*. *faecalis* Symbioflor 1) was cultured overnight in lysogeny broth Luria (LB-Luria). 1 mL bacterial culture was centrifuged at 8000 × g for 5 min to pellet the bacteria. The supernatant was discarded and the pellet was washed three times with sterile saline (0.9% NaCl). 10-fold serial dilutions in sterile saline were prepared and a volume of 100 μL of each dilution was plated onto LB-Luria agar plates and then incubated overnight at 37 °C to count the number of bacteria (CFU). The concentration of *E*. *faecalis* Symbioflor 1 in sterile saline after washing was 6.6 × 10^4^ CFU/μL, and 200 μL aliquots of the bacterial suspension in this concentration were stored at −20 °C until use.

### DNA and RNA template

*E*. *faecalis* Symbioflor 1 genomic DNA and total RNA were extracted using the QIAamp DNA Mini Kit (Qiagen, Hilden, Germany) and the RNeasy Mini Kit (Qiagen, Hilden, Germany), respectively. All extraction procedures were performed according to the manufacturer’s instructions. DNase I (Ambion, Kaufungen, Germany) treatment was used to remove trace amounts of genomic DNA from total RNA. The final concentrations of genomic DNA and total RNA were determined spectrophotometrically using a NanoPhotometer (Implen, München, Germany) which were 17.5 ng/μL and 50.5 ng/μL, respectively.

### Serial dilution of DNA, RNA and bacteria for detection limit determination

The copy number of genomic DNA after extraction was calculated using the following formula: (6.02 × 10^23^) × (ng/μL × 10^−9^)/(DNA length × 660) = copies/μL. The full length of *E*. *faecalis* Symbioflor 1 genomic DNA is about 2.8 × 10^6^ bp. The copy number of genomic DNA after calculation was 5.8 × 10^6^ copies/μL. A 10-fold dilution series from 5.8 × 10^2^ to 5.8 × 10^−1^ copies/μL by EDTA treated WHB was prepared.

The copy number of transfer-messenger RNA (tmRNA) in total RNA was determined by real time RT-PCR of the total RNA and then interpolated to the RNA standard curve which was prepared using real-time RT-PCR of chemical synthetic tmRNA (Biomers, Ulm, Germany). The copy number of tmRNA in total RNA was 6.8 × 10^8^ copies/μL. Four 10-fold dilution series: 4 × , 8 × , 12 × and 16 × (6.8 × 10^5^ to 6.8 × 10^2^ copies/μL) corresponding to 10%, 20%, 30% and 40% WHB by DEPC-treated water were prepared.

A pellet of 6.6 × 10^4^ CFU/μL *E*. *faecalis* Symbioflor 1 was resuspended in 100 μL EDTA treated WHB and a 10-fold dilution series from 6.6 × 10^2^ to 6.6 × 10^−1^ CFU/μL by EDTA treated WHB was prepared.

### Design of primer and probe

Primer pairs and hydrolysis probe used in this study were designed to detect stretches from the *CNRZ16* gene of *E*. *faecalis* Symbioflor 1 which encodes tmRNA using the Primer3plus online service. The primer pair used for the nested real-time PCR reactions was designed from the region within the sequence of the first round PCR and RT-PCR amplicons. Sequences of the primer pairs and hydrolysis probe along with the sizes of the expected amplicons are summarized in Table 1.

**Table 1:** Primer pairs and hydrolysis probe for the quantitative nested real-time PCR and RT-PCR.

	Amplicon length (bp)	Forward primer	Reverse primer	Probe
Outer primer	302	5′-TGAATTGCGTTTCGTAGGTTAC-3′	5′-CCAAACATATTGCCACTTAAATCTC-3′	5′-TCGGGTCAGGGTCCTAATCGAAGTGG-3′
Inner primer	72	5′-CGGCATCGCCCATGTG-3′	5′-ATTTTACAGACGGAAAAATTTAGCG-3′.	

### Quantitative nested real-time PCR and RT-PCR

The quantitative nested real-time PCR comprised a first round PCR and a nested real-time PCR. The quantitative nested real-time RT-PCR comprised a one-step first round RT-PCR and a nested real-time PCR. The first round PCR and RT-PCR reactions were performed on a MJ Research PTC-225 Thermal Cycler (Bio-Rad, München, Germany) with a volume of 40 μL. The nested real-time PCR reactions were performed on a LightCycler 1.5 (Roche, Mannheim, Germany) with a volume of 20 μL. The amplification curves and Cq values of the nested real-time PCR reactions were recorded and analyzed using Roche LightCycler software version 3.5.

The reaction mixtures for the first round PCR and the subsequent nested real-time PCR of the quantitative nested real-time PCR contained 0.5 μM of each primer, 0.2 mM of dNTP mix, 0.25 U/μL *Tth* polymerase (Bioron, Ludwigshafen, Germany) and 1 × PCR buffer: 10 mM Tris-HCl, 0.1 M KCl, 50 μg/mL BSA, 0.05% (w/v) Tween 20, and 1.5 mM MgCl_2_. A separate solution of 100 mM MgCl_2_ (Bioron, Ludwigshafen, Germany) was used for titration in the first round PCR. 0.3 μM hydrolysis probe was used in the nested real-time PCR. Varying amounts of WHB specimens (4, 8, 12, 16 and 20 μL) were added directly to the first round PCR to make up 10–50% of the total reaction volume (the highest concentration of WHB was 56% based on the PCR protocol). 1 μL genomic DNA was added to the first round PCR as the last ingredient and 1 μL of the first round PCR amplicons after 100-fold dilution were used as template in the nested real-time PCR. When varying amounts of blood-DNA mixtures or blood-bacteria mixtures (4, 8, 12, 16 and 20 μL) were used for detection limit determination, 1 μL of the first round PCR amplicons were used directly in the nested real-time PCR without dilution. The first round PCR and the nested real-time PCR were performed at 94 °C for 2 min, followed by 40 amplification cycles at 94 °C for 10 s, 53 °C for 20 s, 72 °C for 30 s, and a final extension at 72 °C for 7 min.

The reaction mixtures for the first round RT-PCR and the subsequent nested real-time PCR of the quantitative nested real-time RT-PCR contained 0.5 μM of each primer, 0.2 mM of dNTP mix, and 0.25 U/μL *Tth* enzyme. The RT-PCR buffer used in the first round RT-PCR was as follows: 50 mM bicine/KOH, 115 mM K-acetate, and 8% glycerol (v/v). The PCR buffer used in the nested real-time PCR was as mentioned above. A separate solution of 100 mM MnCl_2_ (Bioron, München, Germany) was used for titration in the first round RT-PCR. 0.3 μM hydrolysis probe was used in the nested real-time PCR. Varying amounts of WHB specimens (4, 8, 12 and 16 μL) were added directly to the first round RT-PCR to make up 10–40% of the total reaction volume (the highest concentration of WHB was 46.5% based on the RT-PCR protocol). 50 U RNase inhibitor (Ambion, Kaufungen, Germany) was added to the first round RT-PCR mixture and mixed thoroughly by vigorous shaking to inhibit RNases. 1 μL total RNA was added to the first round RT-PCR as the last ingredient and 1 μL of the first round RT-PCR amplicons after 100 times dilution were used as template in the nested real-time PCR. When 1 μL of four total RNA dilution series or varying amounts of blood-bacteria mixtures (4, 8, 12 and 16 μL) were used for detection limit determination, 1 μL of the first round RT-PCR amplicons were used directly in the nested real-time PCR without dilution. The RT step was performed at 61 °C for 30 min, the following first round PCR and the nested real-time PCR were performed as mentioned above. When blood-bacteria mixtures were added to the first round PCR or RT-PCR reactions, the denaturation step at 94 °C was extended to 10 min for improved lysis of the bacteria. Each quantitative nested real-time PCR or RT-PCR was performed in triplicate.

No template control 1 (NTC 1) contained neither WHB nor nucleic acid template. No template control 2 (NTC 2) contained 10% WHB and no nucleic acid template. NTC 3 was PCR detection of total RNA after DNase I treatment. The exogenous DNA added to the first round PCR was ultimately added to the nested real-time PCR after 100-fold dilution independent of the success of the first round PCR, resulting in an amplification curve. Therefore, no cycling control (NCC) was used to indicate the success of the first round PCR. NCC for the quantitative nested real-time PCR contained 10% WHB and 1 μL DNA template and the amplification protocol for NCC only contained the nested real-time PCR. The PCR mixtures for NCC were incubated at room temperature during the first round PCR and 1 μL of these PCR mixtures were used as template in the nested real-time PCR reactions after 100-fold dilution. The definition for the success of the first round PCR was a Cq value of 3 Cq values lower than the mean Cq value for the NCC. The first round PCR and RT-PCR products were visualized on 1.5% agarose gels with gel green staining (Biotium, Hayward, USA). The gels were recorded by the gel documentation system (FujiFilm, Tokyo, Japan).

### Data availability

The datasets generated during and/or analyzed during the current study are available from the corresponding author on reasonable request.

## Electronic supplementary material

Supplementary information
