# Supplementary material for: Direct DNA and RNA detection from large volumes of whole human blood
Source: Sci Rep. 2018 Feb 21;8:3410. doi: 10.1038/s41598-018-21224-0 (PMC5821888; doi:10.1038/s41598-018-21224-0)
Supplement: Supplementary file 1 — Supplementary information [file 41598_2018_21224_MOESM1_ESM.pdf]

# Direct DNA and RNA detection from large volumes of whole human blood

Dongyang Cai[1], Ole Behrmann[1], Frank Hufert[2], Gregory Dame[2,*] and Gerald Urban[1,*]

[1]Department of Microsystems Engineering, University of Freiburg, Freiburg, DE-79110, Germany
[2]Department of Microbiology and Virology, Brandenburg Medical School Fontane, Senftenberg, DE-01968, Germany

Correspondence and requests for materials should be addressed to G.U. (email: gerald.urban@imtek.uni-freiburg.de) or G.D. (gregory.dame@mhb-fontane.de)

**Supplementary Figures:**

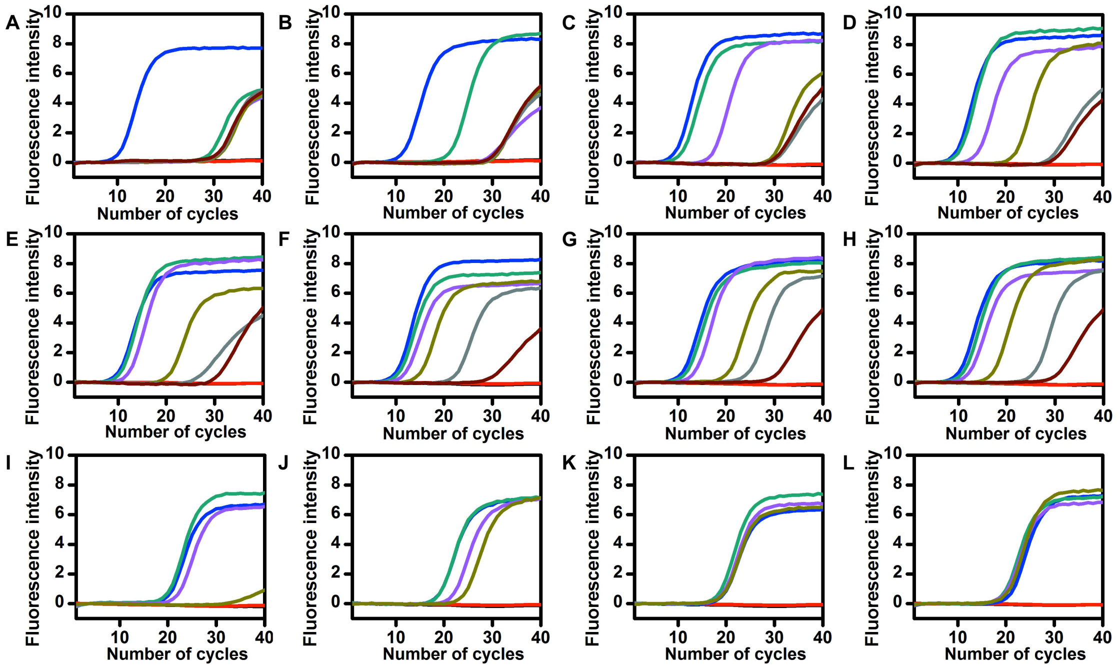


**Figure S1.** The amplification curves for DNA (A-H) and RNA (I-L) detection from varying amounts of EDTA treated WHB in the presence of different concentrations of magnesium ions and manganese ions. From A to H, the concentrations of magnesium ions used in the first round PCR reactions were 1.5, 2, 3, 4, 5, 6, 7 and 8 mM, respectively; from I to L, the concentrations of manganese ions used in the first round RT-PCR reactions were 2, 3, 4 and 5 mM, respectively. The blue, dark cyan, magenta, dark yellow, gray, black, red and wine curves represented 10%, 20%, 30%, 40%, 50% WHB, NTC1, NTC2, and NCC, respectively. NTC 1 and 2 were used to detect the cross-contamination of PCR and RT-PCR reagents and WHB specimens, respectively; NCC was used to indicate the success of the first round PCR reactions.

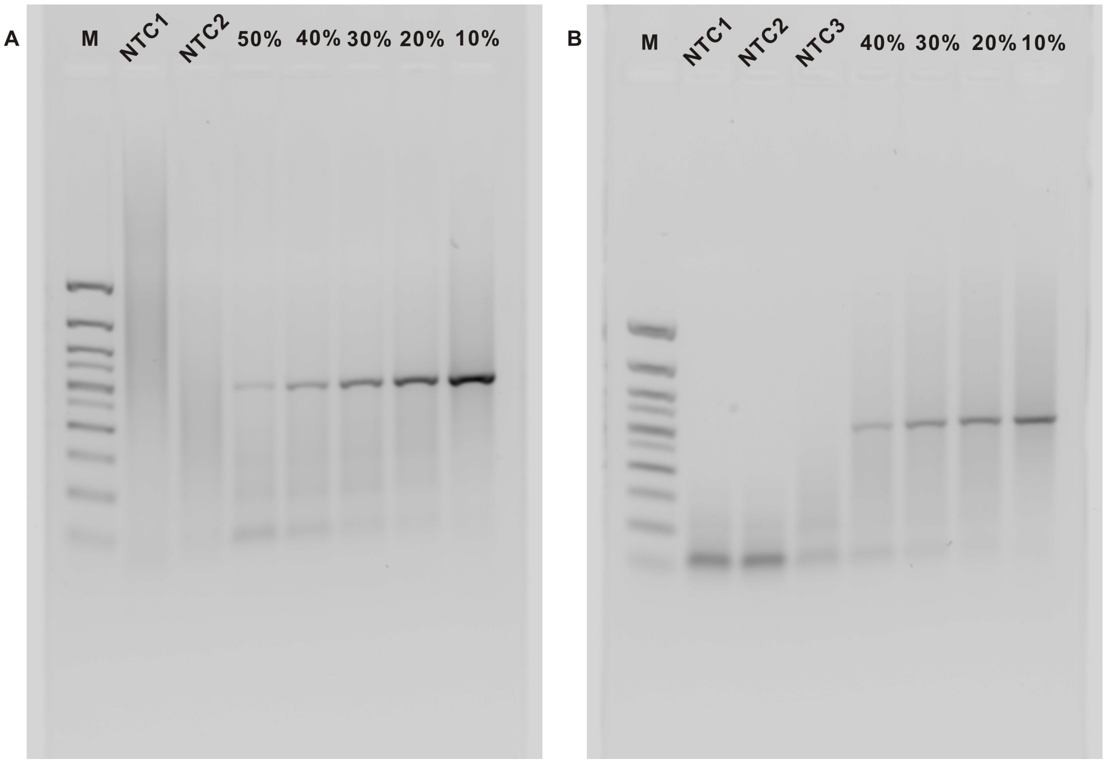


**Figure S2.** The first round PCR for DNA detection from varying amounts of WHB in the presence of 6 mM magnesium ions (A); the first round RT-PCR for RNA detection from varying amounts of WHB in the presence of 4 mM manganese ions (B). M: 50 bp DNA marker; NTC 1 and NTC 2 were used to detect the cross-contamination of PCR and RT-PCR reagents and WHB specimens, respectively; NTC 3 was PCR detection using total RNA after DNase I treatment as template to indicate the removal of genomic DNA. The exposure time for the gels was 1 second.
